# Cardiac calcium dysregulation in mice with chronic kidney disease

**DOI:** 10.1111/jcmm.15066

**Published:** 2020-02-16

**Authors:** Hung‐Yen Ke, Li‐Han Chin, Chien‐Sung Tsai, Feng‐Zhi Lin, Yen‐Hui Chen, Yung‐Lung Chang, Shih‐Ming Huang, Yao‐Chang Chen, Chih‐Yuan Lin

**Affiliations:** ^1^ Division of Cardiovascular Surgery Department of Surgery Tri‐Service General Hospital National Defense Medical Center Taipei Taiwan; ^2^ Department and Graduate Institute of Pharmacology National Defense Medical Center Taipei Taiwan; ^3^ Grade institute of life sciences National Defense Medical Center Taipei Taiwan; ^4^ Institute of Biomedical Sciences Academia Sinica Taipei Taiwan; ^5^ Department of Biochemistry National Defense Medical Center Taipei Taiwan; ^6^ Department of Biomedical Engineering and Institute of Physiology National Defense Medical Center Taipei Taiwan

**Keywords:** calcium homeostasis, CaMKII, chronic kidney disease, electrophysiology, heart failure

## Abstract

Cardiovascular complications are leading causes of morbidity and mortality in patients with chronic kidney disease (CKD). CKD significantly affects cardiac calcium (Ca^2+^) regulation, but the underlying mechanisms are not clear. The present study investigated the modulation of Ca^2+^ homeostasis in CKD mice. Echocardiography revealed impaired fractional shortening (FS) and stroke volume (SV) in CKD mice. Electrocardiography showed that CKD mice exhibited longer QT interval, corrected QT (QTc) prolongation, faster spontaneous activities, shorter action potential duration (APD) and increased ventricle arrhythmogenesis, and ranolazine (10 µmol/L) blocked these effects. Conventional microelectrodes and the Fluo‐3 fluorometric ratio techniques indicated that CKD ventricular cardiomyocytes exhibited higher Ca^2+^ decay time, Ca^2+^ sparks, and Ca^2+^ leakage but lower [Ca^2+^]_i_ transients and sarcoplasmic reticulum Ca^2+^ contents. The CaMKII inhibitor KN93 and ranolazine (RAN; late sodium current inhibitor) reversed the deterioration in Ca^2+^ handling. Western blots revealed that CKD ventricles exhibited higher phosphorylated RyR2 and CaMKII and reduced phosphorylated SERCA2 and SERCA2 and the ratio of PLB‐Thr17 to PLB. In conclusions, the modulation of CaMKII, PLB and late Na^+^ current in CKD significantly altered cardiac Ca^2+^ regulation and electrophysiological characteristics. These findings may apply on future clinical therapies.

## 1 INTRODUCTION

Cardiovascular disease (CVD) is the leading cause of death in patients with chronic kidney disease (CKD).^1^ CKD patients are 2‐6 times more likely to die of a CVD than progress to dialysis.^2^ CVD mortality rate in end‐stage renal failure patients is 10‐ to 30‐fold higher than the age‐matched general population.^3^

The uraemic milieu contains numerous cardiac risk factors that lead to a distinct cardiac pathology termed uraemic cardiomyopathy (UCM).^4^ UCM impairs cardiac performance and causes global cardiac dysfunction, which greatly contributes to the high mortality of the CKD population.^5^ Unique aspects of UCM include left ventricular hypertrophy (LVH), reduced capillary density, fibrosis, and ventricular remodelling; and LVH is the most prevalent characteristic.^6^ One characteristic feature of LVH in UCM is a metabolic remodelling that results in heart failure (HF). The complex pathogenesis of UCM is not clearly understood.

Electrical excitation coupling (EC coupling) in the normal heart involves the interaction of numerous cellular proteins involved in calcium (Ca^2+^) homeostasis.^7^ Increased intracellular Ca^2+^ in cardiomyocytes initiates lethal ventricular tachyarrhythmias, including ventricular fibrillation (VF), in various cardiomyopathies, such as myocardial ischaemia or HF.^8^, ^9^ Intracellular Ca^2+^ overload in cardiomyocytes triggers activity, delayed afterdepolarizations and life‐threatening ventricular tachyarrhythmia.^10^

One of the major pathological changes in cardiomyopathy is dysregulation of intracellular Ca^2+^ homeostasis, which is caused by functional alterations of the proteins involved in Ca^2+^ release and uptake across the sarcolemma and the sarcoplasmic reticulum (SR).^11^ Decreased SR Ca^2+^ ATPase (SERCA) 2a activity was reported in experimental models of CKD in association with the prolongation of diastolic Ca^2+^ transients.^12^, ^13^ Cyclic adenosine monophosphate (cAMP) in human SR regulates the phosphorylation of phospholamban (PLB) via protein kinase A (PKA), which affects SERCA2 activity and Ca^2+^ transport. Phosphodiesterase 3 (PDE3) inhibition potentiates this signalling pathway.^14^ Ca^2+^/calmodulin‐dependent protein kinase II (CaMKII) phosphorylates several Ca^2+^‐handling proteins, including PLB, SERCA, SR Ca^2+^ release channels (Ryanodine receptor, RyR) and L‐type Ca^2+^ channels, which play important roles in the regulation of intracellular Ca^2+^.^15^ Changes in intracellular calcium concentrations ([Ca^2+^]_i_) are the major determinants of cardiac function, but little is known about alterations in [Ca^2+^]_i_ in CKD‐induced cardiomyopathy.

## 2 MATERIALS AND METHODS

### 2.1 Animal experiments

Chronic kidney disease was induced by way of partial nephrectomy (PNx) in C57BL/6J mice as described formerly.^16^, ^17^ The animal care committee of National Defense Medical Center approved all animal experiments (IACUC‐16‐076). Animals (N = 32) were reared in an environment controlled room with unlimited access to deionized water and provender which consisted of Ca 1.00 (wt/wt%), NaCl 0.28 (wt/wt%), Mg 0.22 (wt/wt%) (LabDiet, Richmond, IN, USA). All mice were randomly divided into 2 groups and acclimated to the rearing room (N = 17 for the sham‐operated mice and N = 15 for the PNx‐treated (CKD) mice). Operation of PNx was performed on 6‐month‐old male C57BL/6 mice by selective cauterization of the entire poles of the left kidney by using a Bovie high‐temp cautery with fine tip (Aaron Medical, St. Petersburg, FL, USA). Stage I of PNx with 2‐mm segment around the hilum was left intact. The stage II of PNx was reached by a procedure to remove the right kidney 2 weeks later after the initial PNx. The animals were sacrificed by CO_2_ inhalation 6 months later after the sham operation or PNx. Then, we collected blood and the hearts were dissected for analysis. The concentrations of blood urea nitrogen (BUN) and creatinine of serum (sCr) were detected by using the Jaffe method (Beckman Coulter Synchron LX System; Beckman Coulter Inc).

### 2.2 Echocardiography

A Philips iE33 ultrasound imaging system with a 7‐15 MHz linear array transducer was used to perform echocardiography (Philips Medical Systems). Inhalation of 3% isoflurane was performed to anaesthesia, until animals were sedated and maintained 1% isoflurane during the examination of echocardiography. First of all, it was to obtain 2D left ventricular (LV) short‐axis images. M‐mode was used to measure the thickness of LV wall and dimension of chamber and ejection fraction (EF) at diastole and systole phases. All measurements of echocardiography were averaged for consecutive 5 cardiac cycles.

### 2.3 Electrocardiographic measurements

Under isoflurane anaesthesia (5% for induction and 2% for maintenance), electrocardiograms (ECGs) were recorded from standard lead II limb leads via a bio‐amplifier (ADInstruments), were connected to a ML845 Powerlab polygraph recorder (ADInstruments) and were continuously displayed throughout the experiment in sham or CKD mice.

### 2.4 Mouse ventricle tissue preparation

Sodium pentobarbital (100 mg/kg) was used to anaesthetize Sham and CKD mice with intraperitoneal injection. The heart and lungs were removed by midline thoracotomy. The ventricle was opened in Tyrode's solution which containing 137 mmol/L of NaCl, 11 mmol/L of dextrose, 15 mmol/L of NaHCO_3_, 4 mmol/L of KCl, 2.7 mmol/L of CaCl_2_, 0.5 mmol/L of NaH_2_PO_4_ and 0.5 mmol/L of MgCl_2_,. The ventricle tissue preparation was dissected first and pinned to the base of a tissue bath. The free end of the ventricle tissue preparation was hooked to a Grass FT03C force transducer (Grass Instrument Co.) with a string. Tissue was perfused at 3 mL/min with normal Tyrode's solution which saturated with 97% O_2_ and 3% CO_2_ mixture. The temperature remained constant at 37°C. These preparations were stabilized for 1 hour before the electrophysiological study.

Recording Action potentials (APs) of the ventricle were performed with borosilicate glass microelectrodes which contained 3 mol/L KCl. Preparations of tissue were connected to a WPI model Duo 773 electrometer (World Precision Instruments) with tension of 150 mg. A TDS2000C oscilloscope was used to display both electrical and mechanical events simultaneously (Tektronix) and Gould TA11 recorder (US Instrument Services). Electrical signals were recorded by removing DC coupling first and passed through a low‐pass filter with 10‐kHz cut‐off frequency, then feed to a Digidata 1320 acquisition system (Molecular Devices). Electrical stimulation was provided with a S88 stimulator with a SIU5B isolation unit (Grass Instrument). Spontaneous activity was defined as occurrence of spontaneous AP with no electrical stimuli. Early afterdepolarization (EAD) was defined as abnormal depolarization occurred with phase 2 or 3 of the APs. APs were evoked by a 4‐Hz electrical stimulus before and after administration of medicine. The RMP was obtained during the phase between the last repolarization and subsequent onset of the AP. The amplitude of action potential (APA) was obtained from the RMP to the apex of the AP depolarization. The action potential duration (APD) at 90% (APD_90_), 50% (APD_50_) and 20% (APD_20_) of the APA were measured accordingly. Data acquisition was performed by using a Digidata 1320 interface (Axon Instruments Inc).

### 2.5 Isolation of single control and uraemic cardiomyocytes

Mice used in this study were anaesthetized by using sodium pentobarbital (100 mg/kg, i.p.). The heart and lungs were removed quickly after a midline thoracotomy was performed. The heart was perfused in a retrograde manner via a dispensing needle (OD, 0.45 mm) cannulated through the aorta and left ventricle into the left atrium. The free end of the dispensing needle was connected to a Langendorff perfusion apparatus and perfused with oxygenated normal Tyrode's solution at 37°C (containing 137 mmol/L of NaCl, 11 mmol/L of glucose, 10 mmol/L of HEPES, 5.4 mmol/L of KCl, 1.8 mmol/L of CaCl_2_ and 0.5 mmol/L of MgCl_2_; pH was adjusted to 7.4 by titrating with 1 N NaOH). The perfusate was replaced with oxygenated Ca^2+^‐free Tyrode's solution which containing 150 units/ml collagenase (Type I, Sigma) and 0.25 units/ml protease (Type XIV, Sigma) for 5‐8 min. The ventricle was separated from the atrium and lung and placed in a chamber which containing Ca^2+^‐free oxygenated Tyrode's solution. Dice the tissue and shaken gently in 5‐10 mL of Ca^2+^‐free oxygenated Tyrode's solution until single cardiomyocytes were obtained. The Ca^2+^‐free solution was changed gradually to oxygenated normal Tyrode's solution.

### 2.6 Intracellular Ca^2+^ regulation

Isolated cardiomyocytes were loaded with 10 μmol/L of fluo‐3/AM for 30 min at room temperature (RT). The bath solution was changed to remove excess dye at 35 ± 1°C for 30 minutes. A 488‐nm argon ion laser was used to excite Fluo‐3. The emission fluorescence was recorded at >515 nm. Cells were scanned repetitively at 3‐ms intervals for a total duration of 6 seconds. Fluorescence imaging was performed using a confocal microscope (Leica TCS SP5). Signals were corrected by dye concentration via the normalization of the fluorescence (F) against the baseline fluorescence (F_0_) in order to obtain data of intracellular Ca^2+^ transient [Ca^2+^]_i_ changes from baseline values (△F/F_0_), and exclude fluctuations in the fluorescence intensity with different volumes of injected dye. Calcium transients at peak systolic and diastolic activities were measured by 1‐Hz field stimulation with 10‐ms twice‐threshold strength square‐wave pulses. The Ca^2+^ content of SR was measured by adding of 20 mmol/L of caffeine with pacing at 1 Hz for at least 30 seconds. All measurements were performed at a temperature of 35 ± 1°C. Background subtraction was also performed for fluorescence measurement.

### 2.7 Calcium sparks

Calcium sparks were detected by aligning the scanning line along a line parallel to the longitudinal axis of single control and uraemic ventricular cardiomyocytes to avoiding the interference of nuclei. Each scanning line is composed of 512 pixels. Calcium sparks were analysed by using SparkMaster and validated by authors performed with criteria of the signal mass.^18^ Calcium spark frequencies were expressed as the number of sparks observed (per second and per μm of distance scanned with), and the incidences were expressed as the percentage of cells showing Ca^2+^ sparks during the diastolic phase.

### 2.8 Western blot analysis

Lysis buffer (Tris‐Cl 1 mol/L PH adjusted to 8.0, NaCl 1.2 mol/L, Nonidet P‐40 0.5% v/v) containing 10 μL/mL of a protease inhibitor (Sigma) was used to homogenized tissue for 15 minutes on ice, followed by 14 000 *g* centrifugation for 15 minutes at 4°C. Samples were then boiled with 2‐mercaptoethanol (2MP, 5 μL/100 mL) for 5 minutes to reducing sample buffer and then transferred to 10% SDS‐PAGE. Sample proteins were transferred to nitrocellulose membranes which were blocked at 4°C in 5% milk in PBS with 0.01% Tween‐20 (PBST) overnight. These nitrocellulose membranes were incubated with diluted primary antibodies for 1 hour at RT. Then, washing them in PBST for 15 minutes and repeat 3 times. Membranes were incubated in diluted (1:10 000) secondary horseradish peroxidase (HRP)‐conjugated antibodies for 1 hour, then washed thoroughly in PBST for 15 minutes (repeat for 3 times). ECL Plus Western blotting detection reagents (Amersham Biosciences) and X‐ray film (Eastman Kodak) were used to visualize bands. The following primary antibodies were diluted in BSA: rabbit anti‐RyR (Abcam, UK), mouse anti‐SERCA2 (Santa Cruz Biotechnology), rabbit anti‐phosphorylated RyR (pRyR, ab59225, Abcam), rabbit anti‐phosphorylated SERCA2 (pSERCA2, A010‐25AP, Badrilla), PLB phosphorylated at Thr17 (PLB‐Thr17) (Badrilla), phosphorylated CaMKII at Thr 286 (pCaMKII), total PLB (Thermo), CaMKII and NCX (Swant). A GAPDH antibody was used to normalize protein bands in each blot. ImageJ software was used to quantify relative protein level.

### 2.9 Statistical analysis

All quantitative data are expressed as the means ± the standard error of the mean (SEM). Statistical significance between different groups was determined by an unpaired *t* test or one‐way analysis of variance (ANOVA) with Tukey's test for multiple comparisons, as appropriate. A value of *P* < .05 was considered significant.

## 3 RESULTS

### 3.1 Cardiac structure, functions, and ECGs of sham and CKD mice

**Figure 1:**
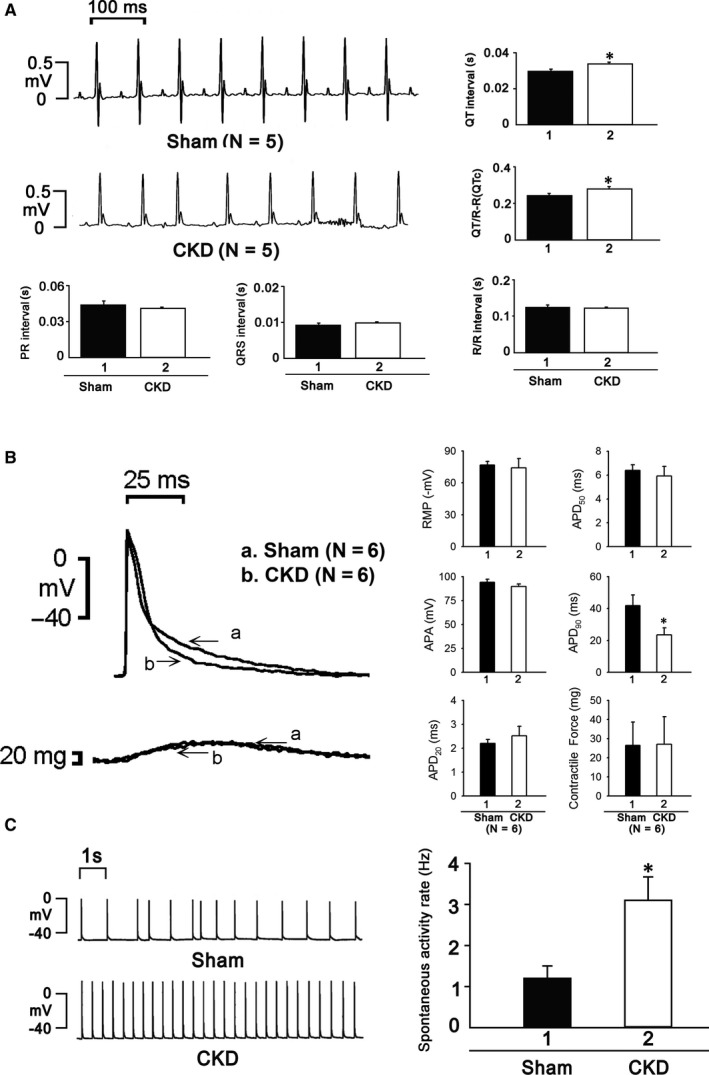
A, ECGs showed prolongation of QT interval and QTc, but not RR interval, in CKD mice. B, APD_20_, APD_50_ and contractile force were similar in both groups, but APD_90_ was significantly shorter in CKD mice. C, The heart beats of CKD mice were significantly higher

As shown in Table 1, significantly increased serum BUN (49.0 ± 12.64 mg/dL vs 18.9 ± 0.51 mg/dL, *P* < .05) and creatinine levels (1.24 ± 0.36 mg/dL vs 0.45 ± 0.01 mg/dL, *P* < .05) in CKD mice confirmed the successful induction of experimental renal failure. The heart rate and left ventricular mass in the sham and CKD mice were not significantly different. CKD mice exhibited a greater left atrium diameter to aortic root diameter ratio (LA/AO), left ventricular internal diameter at end‐diastole (LVIDd), left ventricular internal diameter at end‐systole (LVIDs), end‐diastolic volume (EDV) and end‐systolic volume (ESV) compared with the sham mice. The fractional shortening (FS, 23.1 ± 2.6% vs 32.4 ± 2.5%, *P* < .05) and stroke volume (SV, 0.082 ± 0.003 ml vs 0.103 ± 0.004 ml, *P* < .05) were decreased in CKD mice compared to sham mice. The ECG data showed that CKD mice exhibited longer QT intervals and corrected QT (QTc) prolongation than the sham group (Figure 1A). However, the RR intervals were similar in sham and CKD groups (Figure 1A).

**Table 1:** Summary of heart rate, left ventricular mass, serum blood urea nitrogen (BUN), creatinine and echocardiography measurements

	Sham (N = 11)	CKD (N = 15)
HR (min)	483.5 ± 19.7	460.0 ± 14.6
LV mass (g)	0.105 ± 0.012	0.095 ± 0.011
BUN (mg/dL)	18.9 ± 0.51	49.0 ± 12.64^a^
Creatinine (mg/dL)	0.45 ± 0.01	1.24 ± 0.36^a^
LA/AO	1.35 ± 0.08	1.5 ± 0.08
LVIDd (mm)	0.384 ± 0.013	0.4 ± 0.013
LVIDs (mm)	0.26 ± 0.016	0.31 ± 0.02
EDV (mL)	0.142 ± 0.014	0.162 ± 0.015
ESV (mL)	0.05 ± 0.007	0.08 ± 0.014
FS (%)	32.4 ± 2.5	23.1 ± 2.6^a^
SV (mL)	0.103 ± 0.004	0.082 ± 0.003^a^

Note

BUN and creatinine levels were increased in CKD mice. The fractional shortening and stroke volume decreased significantly in CKD mice. CKD, chronic kidney disease; HR, heart rate; LV, left ventricular; BUN, blood urea nitrogen; LA/AO, left atrial to aortic root ratio; LVIDd, left ventricular internal diameter at end‐diastole; LVIDs, left ventricular internal diameter at end‐systole; EDV, end‐diastolic volume; ESV, end‐systolic volume; FS, fractional shortening; SV, stroke volume. Values are expressed as the means ± SEM.

a *P* < .05 compared to sham mice.

The electrophysiological experiments showed that ventricular cardiomyocytes of CKD mice exhibited significantly shorter APD_90_ but similar APD_20_, APD_50_ and contractile forces as the sham group (Figure 1B). The heart beating rate was significantly higher in CKD mice (Figure 1C).

### 3.2 Effects of CKD on Ca^2+^ regulation

**Figure 2:**
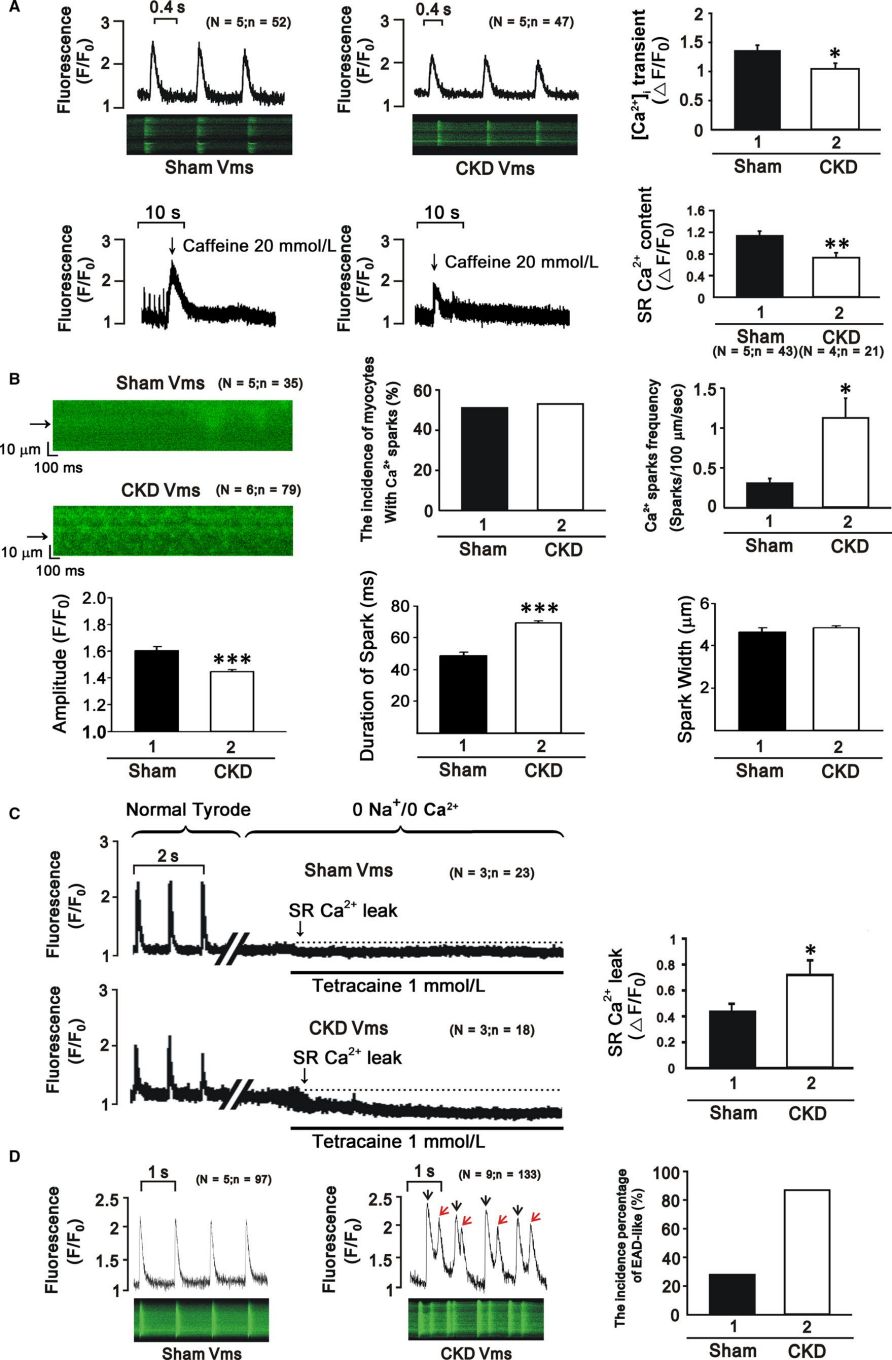
A, CKD ventricular myocytes exhibit lower Ca^2+^ transients and decreased SR Ca^2+^ content. B, The incidence and frequency of Ca^2+^ sparks increased unevenly in cardiomyocytes of CKD mice. C, The ratio of Ca^2+^ leakage increased in CKD mice. D, More EADs were observed in CKD mice

CKD ventricular myocytes exhibited lower Ca^2+^ transients than sham ventricular myocytes and decreased SR Ca^2+^ content (Figure 2A). Ca^2+^ sparks are primary Ca^2+^ release from the stochastic opening of one or more RyRs in cardiomyocytes, and unusual Ca^2+^ spark dynamics are involved in various pathologies, such as heart failure and cardiac arrhythmia.^19^, ^20^, ^21^ The incidence and frequency of Ca^2+^ sparks increased unevenly in cardiomyocytes of the CKD group (Figure 2B). The ratio of Ca^2+^ leakage increased in CKD ventricular myocytes (Figure 2C).

Early afterdepolarization (EAD) is defined as interruption of phase 2 or 3 of action potentials prior to full repolarization, and EADs are related to a shortening of action potential durations (APDs).^22^ Our study demonstrated a greater occurrence of EAD‐like waves in the right ventricles of CKD mice (Figure 2D).

### 3.3 Protein expression levels of Ca‐regulatory proteins in ventricular cardiomyocytes

**Figure 3:**
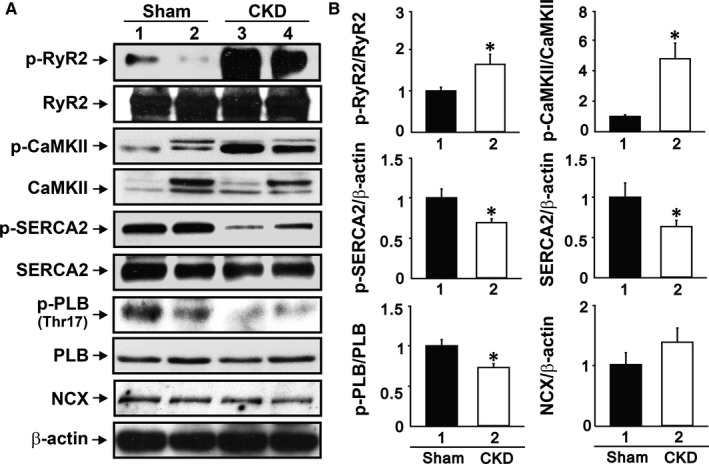
CKD mice exhibit a higher ratio of phosphorylated RyR2 and CaMKII. Phosphorylated SERCA2, SERCA2 and the ratio of PLB‐Thr17 to PLB were reduced in CKD mice. The expression of NCX was similar in both groups

CKD ventricles exhibited higher phosphorylation of RyR2 and CaMKII than the sham groups (Figure 3). The protein levels of p‐SERCA2 and SERCA2, and the ratio of PLB‐Thr17 to PLB was reduced in CKD right ventricles. However, CKD right ventricles exhibited similar levels of NCX compared with the sham groups.

### 3.4 Effects of KN93 and RAN treatment on electrophysiological characteristics

**Figure 4:**
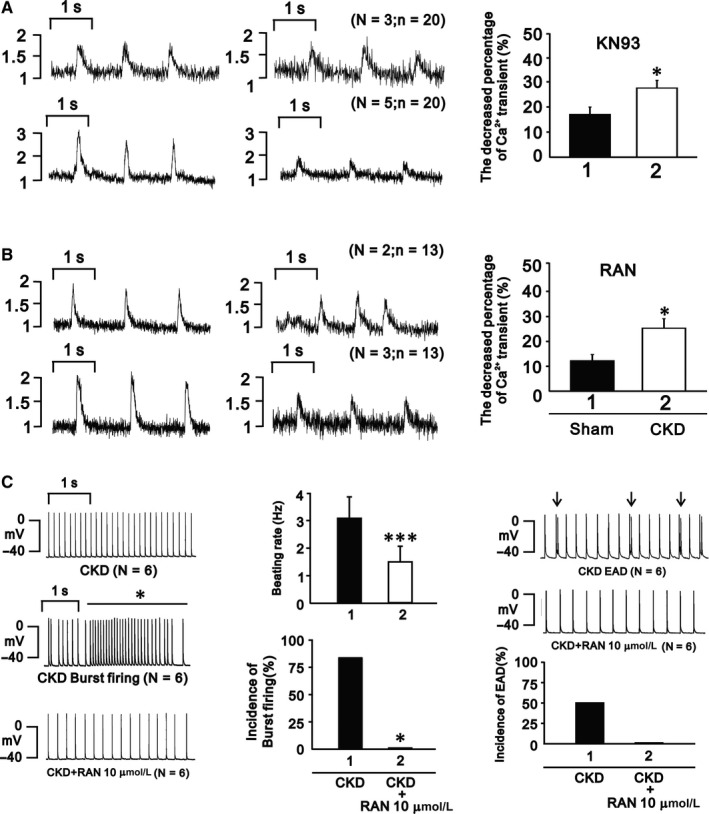
A and B, KN93 and RAN significantly decreased Ca^2+^ transients in CKD mice. C, RAN reversed the alterations of heart beat, incidence of burst firing, and EADs in CKD mice

The addition of KN93 (CaMKII inhibitor) and RAN (late sodium current inhibitor) to CKD and sham cardiomyocytes produced a significant decrease in Ca^2+^ transients in the CKD group but little effect in the sham group (Figure 4A,4). RAN addition to CKD cardiomyocytes significantly decreased the beating rates, incidence of burst firing and the incidence of EADs (Figure 4C).

## 4 DISCUSSION

We successfully created an animal model of CKD‐inducing heart failure in mice and investigated the role of inflammation on heart failure progression.

### 4.1 Ca^2+^ handling in cardiomyocytes of CKD mice

Ca^2+^ is a major ionic modulator of the heart, and it plays an important role in the excitation‐contraction coupling process. Ca^2+^‐induced Ca^2+^ release (CICR) describes a biological process whereby Ca^2+^ activates the release of further ionic Ca^2+^ from SR stores via the RyR to increase [Ca^2+^]_i_ and aid in the binding of Ca^2+^ to myofilaments in the initiation of cardiac contraction.^23^ Our results showed that the ventricular myocytes of CKD mice exhibited decreased Ca^2+^ transients, prolonged transient decay, increased Ca^2+^ leak and decreased SR Ca^2+^ content, which decreased fractional shortening and was arrhythmogenic with more EADs.

CaMKII plays multiple roles in cellular ionic regulation. CaMKII inhibition diminishes cardiac arrhythmias in vitro and in vivo, and transgenic CaMKII‐overexpressing mice exhibit an increased incidence of cardiac arrhythmogenesis.^24^ The present study observed higher CaMKII protein expression in CKD ventricular tissue. A recent study discovered that protein kinase A (PKA) phosphorylated PLB‐Ser16 and CaMKII exclusively catalysed the phosphorylation of PLB‐Thr17.^25^ Our study demonstrated increased phosphorylation of PLB‐Thr17 protein and no change in PLB‐Ser16 phosphorylation in CKD ventricles (data not shown), which suggests higher CaMKII, not PKA, activity in CKD ventricles. CaMKII‐enhanced diastolic Ca^2+^ leakage also hyperphosphorylates RyR, which leads to early afterdepolarizations.^26^ Ca^2+^ sparks represent a major release of Ca^2+^ in cardiomyocytes during excitation‐contraction coupling.^19^ Ca^2+^ sparks are stochastic activations of a cluster of RyR2s that are organized into a Ca^2+^‐release unit.^27^ Diabetes mellitus rats exhibit a higher incidence and frequency of Ca^2+^ sparks in cardiomyocytes,^28^ which leads to changes in Ca^2+^ handling and myocardial dysfunction. We also found an increased frequency and incidence of Ca^2+^ sparks in CKD mice. Thus, the increased Ca^2+^ leakage from the SR may produce a Ca^2+^ deficiency, which leads to myocardial dysfunction in CKD cardiomyopathy. These findings suggest that increased CaMKII in CKD ventricles was arrhythmogenic, and the increased SR Ca^2+^ leak was a crucial mechanism, which is consistent with our findings.

A review from Harvath and Bers found that the increased late sodium current also contributed to Ca^2+^ modulation to cause heart failure, which was associated with increased ROS.^29^ Our data showed that RAN treatment abrogated CKD‐affected Ca^2+^ transient, decay time and AP, which suggests that a deterioration of Na^+^ regulation also modulated intracellular Ca^2+^ handling in CKD.

### 4.2 ECG changes in CKD

CKD patients exhibit a high incidence of cardiovascular complications that are characterized by complex alterations in the mechanical and electrical properties of the heart.^30^ Previous studies of CKD patients revealed prolongation of QT and QTc intervals.^31^ Our study found that the CKD mice exhibited prolonged QT and QTc intervals, but there was no effect on RR interval duration. QT records the sum of millions of individual APDs from both right and left ventricle, and could conceal within itself a number of short APDs juxtaposed to long ones. In our study, CKD shortened APD_90_ but prolonged QT and QTc intervals. We only recorded APDs from right ventricle, but not from left ventricle. It is possible that the prolongation of QT and QTc intervals may be contributed mostly by prolonged APDs from left ventricle. Therefore, the discrepant effects of CKD on APDs between right and left ventricle may increase interventricular dispersion of APDs, which could increase the genesis of micro‐reentry circuits.^32^ Moreover, prolonged QT interval may result in ventricular arrhythmia due to triggered activity of EAD,^33^ and we found more EADs in our CKD mice.

### 4.3 The phosphorylation of RyR2, SERCA2 and PLB may depend on the histological type of tissue

The CKD pulmonary vein (PV) cardiomyocytes of rabbits exhibit higher SR Ca^2+^ contents and calcium transient amplitudes, which may be due to the enhanced phosphorylation of PLB or RyR2 and SERCA2a activity.^34^ KN93 (1 μmol/L) eliminated these effects.^34^ Our study found that ventricular cardiomyocytes from CKD mice exhibited decreased SR Ca^2+^ contents and Ca^2+^ transient amplitudes than sham mice. RyR2 phosphorylation levels increased, but SERCA2 and PLB phosphorylation decreased. These distinct findings suggest a different remodelling of Ca^2+^‐regulating proteins at different sites in cardiomyocytes, which produces arrhythmogenic or contractile dysfunction and heart failure.

## 5 CONCLUSIONS

**Figure 5:**
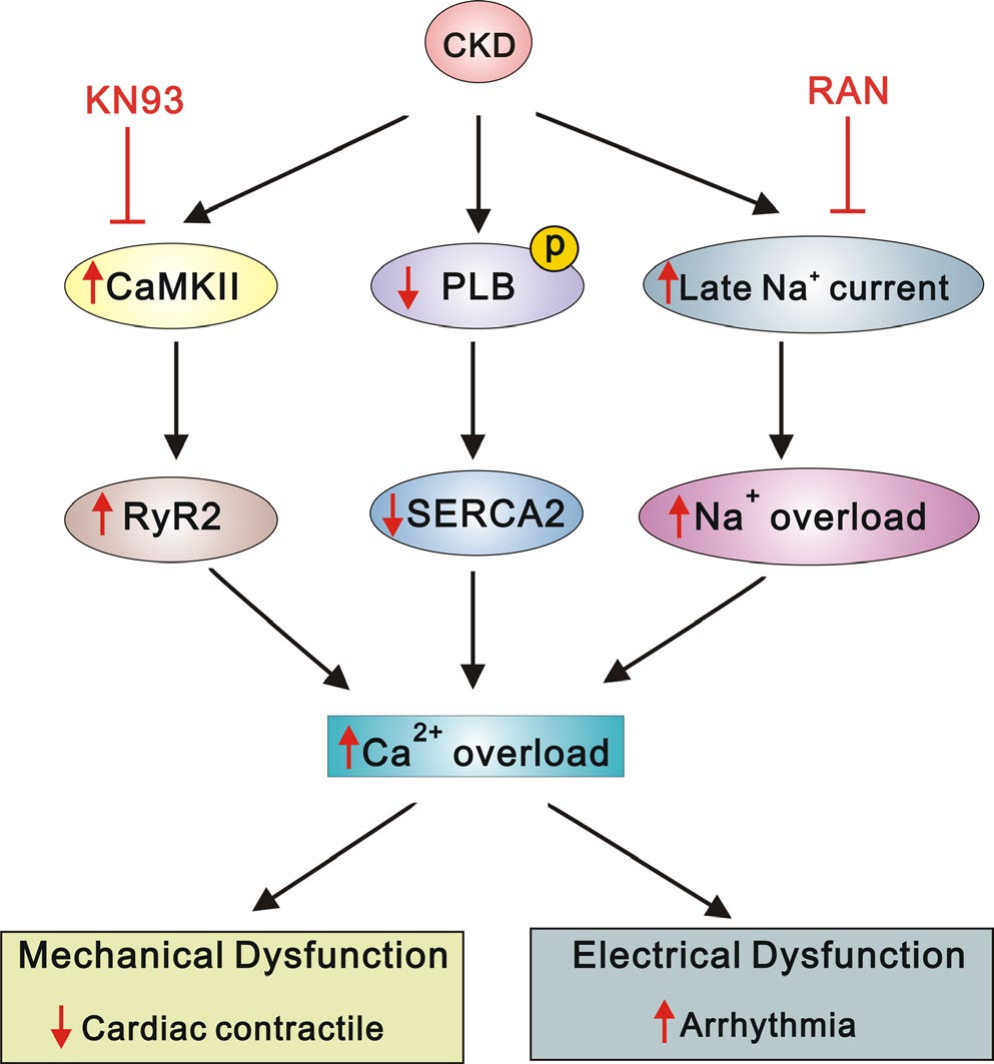
The proposed mechanism of calcium dysregulation in uraemic cardiomyopathy. The underlying mechanisms were associated with the regulation of CaMKII, PLB and late Na current

Our findings suggest that CKD induces uraemic cardiomyopathy, which exhibits alterations in cardiac Ca^2+^ regulation and electrophysiological characteristics (Figure 5). These alterations are associated with the regulation of CaMKII, PLB and late Na^+^ current. The antagonists KN93 and RAN may eliminate the observed effects.

## CONFLICT OF INTEREST

All authors declare that they have no competing interests.

## AUTHORS CONTRIBUTIONS

HYK, LHC and CYL devised the project, the main conceptual ideas. CST and CYL performed the numerical calculations for the experiment and revised the manuscript. LHC, FZL, YHC and HYK performed the measurements, SMH and YCC were involved in supervised the work, HYK and YCC processed the experimental data and performed the analysis. HYK drafted the manuscript and designed the figures. YLC and LHC performed the excel calculations. CST, SMH, YCC and CYL aided in interpreting the results. All authors discussed the results and commented on the manuscript.

## Data Availability

The data that support the findings of this study are available from the corresponding author upon reasonable request.
